# The “Black Pattern”, a Simplified Ultrasound Approach to Non-Traumatic Abdominal Emergencies

**DOI:** 10.3390/tomography8020066

**Published:** 2022-03-15

**Authors:** Stefania Tamburrini, Letizia Consoli, Marco Garrone, Giuseppe Sfuncia, Marina Lugarà, Maria Gabriella Coppola, Miryam Piccirillo, Roberta Toto, Salvatore Massimo Stella, Soccorsa Sofia, Mario Scuderi, Orlando Catalano

**Affiliations:** 1 Department of Radiology, Ospedale del Mare-Asl NA1 Centro, Via Enrico Russo, 11, 80147 Napoli, Italy; 2 Advanced Emergency Ultrasound SIUMB School, 80147 Naples, Italy; marinalugara82@gmail.com (M.L.); gabry.cop@libero.it (M.G.C.); piccirillo.mym@hotmail.it (M.P.); toto.roberta@gmail.com (R.T.); orlando.catalano@istitutovarelli.it (O.C.); 3 Department of Emergency Medicine, Fondazione Poliambulanza, Via Bissolati 57, 25124 Brescia, Italy; letizia.consoli@icloud.com; 4 Advanced Emergency Ultrasound SIUMB School, 25124 Brescia, Italy; 5 Department of Emergency Medicine, A.O. Mauriziano, Largo Filippo Turati, 62, 10128 Torino, Italy; marcogarrone@gmail.com; 6 Advanced Emergency Ultrasound SIUMB School, 10128 Torino, Italy; 7 Department of Emergency Medicine, Ospedale santi Giovanni e Paolo, Sestriere Castello, 6777, 30122 Venezia, Italy; atropina5mg@gmail.com; 8 Department of Internal Medicine, Ospedale del Mare-Asl NA1 Centro, Via Enrico Russo, 11, 80147 Napoli, Italy; 9 Department of Anesthesiology and Intensive Care, Ospedale del Mare-Asl NA1 Centro, Via Enrico Russo, 11, 80147 Napoli, Italy; 10 Department of Clinical and Experimental Medicine, University of Pisa, Santa Chiara University Hospital, Via Roma, 67, 56100 Pisa, Italy; stellasalvatoremassimo@gmail.com; 11 Advanced Emergency Ultrasound SIUMB School, 56100 Pisa, Italy; 12 Department of Emergency Medicine, Ospedale Maggiore-C. A. Pizzardi, Largo Nigrisoli, 2, 40133 Bologna, Italy; sasaxina@gmail.com; 13 Advanced Emergency Ultrasound SIUMB School, 40133 Bologna, Italy; 14 Policlinico Morgagni, Via del Bosco, 105, 95125 Catania, Italy; mscuderi@tiscali.it; 15 Advanced Emergency Ultrasound SIUMB School, 95125 Catania, Italy; 16 Istituto Diagnostico Varelli, Via Cornelia dei Gracchi, 65, 80126 Naples, Italy

**Keywords:** emergency ultrasound, abdominal ultrasound, ultrasound, abdomen, emergency

## Abstract

Background: A key issue in abdominal US is the assessment of fluid, which is usually anechoic, thus appearing “black”. Our approach focuses on searching for fluid in non-traumatic patients, providing a new, simplified method for point-of-care US (POCUS). Objective: Fluid assessment is based on a three-step analysis that we can thus summarize. 1. Look for black where it should not be. This means searching for effusions or collections. 2. Check if black is too much. This means evaluating anatomical landmarks where fluid should normally be present but may be abnormally abundant. 3. Look for black that is not clearly black. This means evaluating fluid aspects, whether wholly anechoic or not (suggesting heterogeneous or corpusculated fluid). Discussion: Using this simple method focused on US fluid presence and appearance should help clinicians to make a timely diagnosis. Although our simplified, systematic algorithm of POCUS may identify abnormalities; this usually entails a second-level imaging. An accurate knowledge of the physio–pathological and anatomical ultrasound bases remains essential in applying this algorithm. Conclusion: The black pattern approach in non -traumatic emergencies may be applied to a broad spectrum of abnormalities. It may represent a valuable aid for emergency physicians, especially if inexperienced, involved in a variety of non-traumatic scenarios. It may also be a simple and effective teaching aid for US beginners.

## 1. Introduction

Ultrasound (US) is rightly regarded as the initial imaging tool for the evaluation of patients with abdominal pain. It is crucial in the management of both trauma [1,2] and non-traumatic emergencies [3,4].

POCUS is primarily used to answer focused clinical questions at the bedside, to narrow the differential diagnosis and direct early appropriate therapy. This approach has many advantages over traditional imaging modalities: it is rapid, portable, non-invasive, repeatable, less expensive, and does not entail ionizing radiation exposure. POCUS has several applications, including diagnostic, screening, and procedural, as already recognized by leading scientific societies [1,3,5]. Scanning is limited to the areas of interest and focused targets, based on pre-test probability.

Despite the high accuracy reported for POCUS protocols among radiologists and non-radiologists alike, these diagnostic tools are usually limited to confirming or excluding a single clinical hypothesis on which the exam is focused. In such a mindset, the abdomen would not be considered as a whole with each organ or structure representing an independent topic and thus a separate exam. As has already been done for POCUS in traumatic cases, where focus is on free-fluid detection, the purpose of our pattern approach is to apply the search for abdominal fluid in non-traumatic emergencies, considering the abdomen as a whole. Our re-reading of the POCUS approach consists of a three-step analysis. 1. Looking for black where it should not be, 2. looking to see if there is too much black, i.e., if it is overabundant where normally present, 3. looking for black that is not clearly black. On the basis of this simplified US analysis, a first diagnostic hypothesis can thus be proposed, and patients quickly stratified. It can be considered as a first diagnostic approach to acute, non-traumatic abdominal pain, easily performed by all physicians involved in acute care settings. US is widely available nowadays and fluid semeiotics is the first teaching point in the US learning process [6]. Providing basic knowledge of US anatomy and US features of abnormality, the black pattern may represent a quick way to categorize US findings in a single patient and narrow down the list of potential causes.

## 2. Black: US Features

Fluids, especially if poor in cells, appear as black, hypoechoic to anechoic in US, because most of the US’ beam goes through them and only a small part of the waves is reflected and returns to the transducer. The search for black fluid and its possible shades of gray is the basic principle of our POCUS protocol [3,4,7]. This US approach is versatile and a valuable tool for physicians involved in emergency settings and also suitable for physicians without extensive training or background in clinical sonography [8]. The three key steps of the US investigation are summarized below.

### 2.1. Looking for Black Where It Should Not Be

FAST has become the common initial screening modality in most traumas. The goal of FAST examination is the detection of free fluid in the peritoneal cavity, pericardium, and pleural spaces. The volume of fluid accumulation required for visualization by US ranges between 250 and 620 mL [1,2]. Similar to the FAST examination, we propose a first level scan looking for black areas in non-traumatic patients in the abdominal cavity and between the bowel loops or close to the surgical site, and in soft tissue in post-operative patients (Table 1).

The presence of free fluid in non-traumatic patients is highly indicative of a broad spectrum of abnormalities that require prompt diagnosis and management. Patient management often requires a second level of imaging (i.e., contrast-enhanced CT scan) in many cases to identify and stage the pathology and then choose optimal treatment, including medical therapy, intervention, or surgery [4].

Here, we summarize the main etiologies underlying those conditions in which there is black where it should not be.

#### 2.1.1. Non-Traumatic Hemoperitoneum

**Figure 1:**
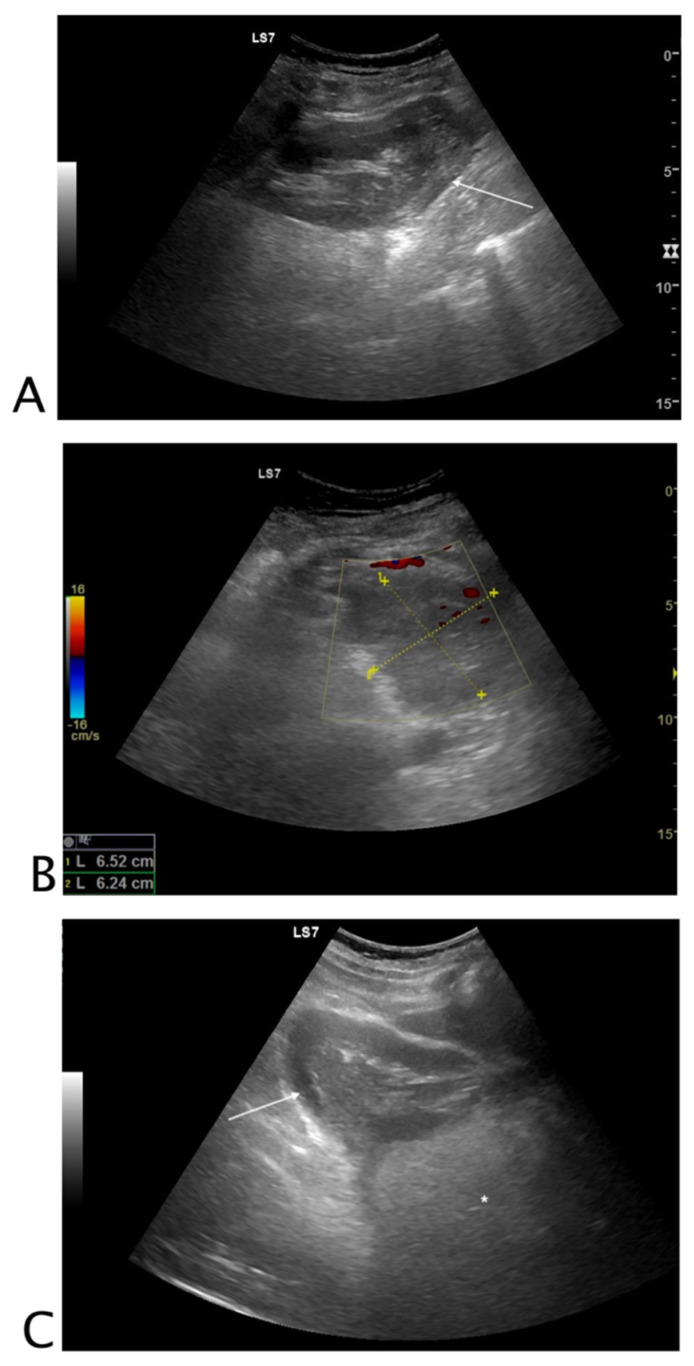
Black where it should not be. A 26-year-old female patient with abdominal tenderness and fainting. At ultrasound, an inhomogeneous subcapsular fluid collection (white arrow) was detected all around the right kidney (**A**). On the lower pole of the kidney, fluid collection was detected (**B**). On the upper pole of the right kidney, a hyperechoic mass (*) was seen, referable to angiomyolipoma (**C**). A suspected ultrasound diagnosis of hemo-retroperitoneum for spontaneous angiomyolipoma bleeding was formulated. The patient underwent CT angiography that confirmed active bleeding of the right kidney’s upper pole angiomyolipoma. The patient underwent endovascular arterial embolization. Final diagnosis: spontaneous bleeding of renal angiomyolipoma.

Identifying the cause of intra-abdominal bleeding in non-traumatic patients is a challenging diagnosis. Causes of non-traumatic hemoperitoneum have been grouped into etiologies such as iatrogenic (related to prior surgery, intervention, or anticoagulation), tumor-related, gynecological, and vascular. The three most common causes among women and in order of decreasing frequency are a ruptured ovarian cyst (women < 35 years old), recent procedure, and unknown mass [5] (Figure 1). The US aspect of hemoperitoneum is free fluid; its appearance is non-specific, it may be hypo-, iso-, or hyperechoic and may demonstrate fluid-fluid level with mixed internal echogenicity. This is mainly related to the onset time of bleeding and proximity of the hemorrhagic source [5,6].

#### 2.1.2. Non-Traumatic Free Fluid

In patients with abdominal distension, US can quickly determine whether abdominal ascites is the cause. The technique is similar to that used in the FAST protocol. The fluid is usually anechoic; however, the presence of free-floating echogenic particles may be telling of exudate or blood [1,4,7,8].

#### 2.1.3. Post-Surgical Complications

**Figure 2:**
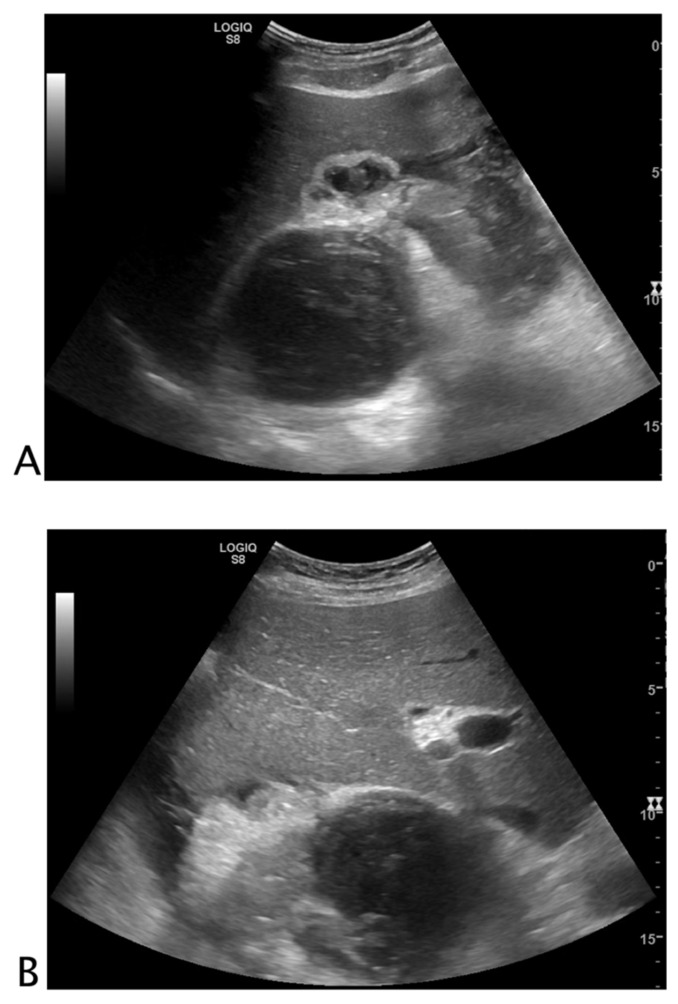
Black where it should not be. A 72-year-old male patient with abdominal pain and malaise after right nephrectomy. The right renal lodge was occupied by loculated fluid collection with fluid/fluid levels and clot (**A**). Perihepatic free fluid with inhomogeneous clot was detected (**B**). A suspected ultrasound diagnosis of post-surgical bleeding was formulated. The patient underwent CT angiography that confirmed the bleeding, and arterial endovascular embolization. Final diagnosis: post-nephrectomy bleeding.

The identification of post-surgical intra-abdominal fluid collections is a challenging task. In post-surgical patients, free fluid or organized loculated fluid collections may be a sign of complications such as ascites, seroma, hematoma, biloma, lymphocele, anastomotic leaks, or abscesses (Figure 2) [9].

Instead, if we focus on the anatomical site where we find anechoic liquid not normally present, we can encounter the following US scenarios:

##### Bowel

**Figure 3:**
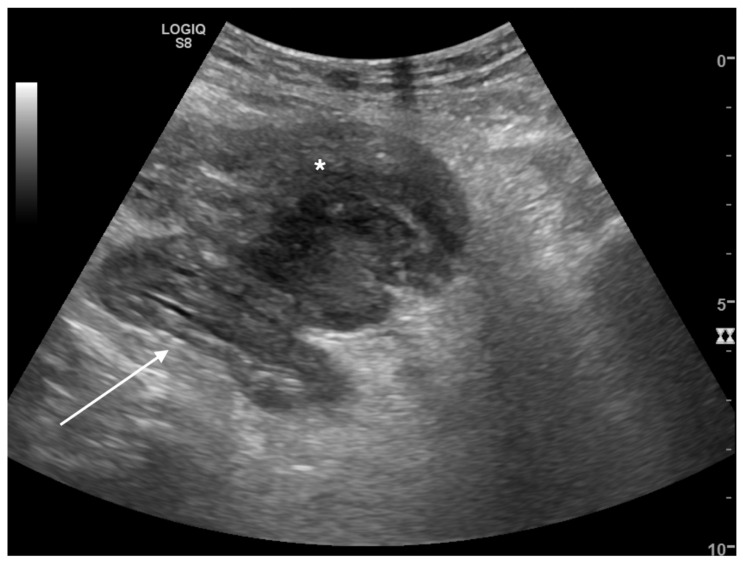
Black where it should not be. A 62-year-old male patient with left lower quadrant abdominal pain. Sigmoid colon appeared stratified with muscular prevalence (white arrow) and perivisceral fat was markedly hypoechogenic (*). Adjacent to the bowel wall, a loculated inhomogeneous fluid collection was detected. A suspected diagnosis of complicated diverticulitis was formulated. Final diagnosis: Hinchey stage II diverticulitis (pelvic abscess > 4 cm).

US cannot determine the presence of visceral perforation directly [10]. Bowel perforation, including post-procedural endoscopic perforation, can be suspected on the basis of the presence of perivisceral fluid or fluid collection with air bubble inclusions adjacent to a thickened bowel segment (Figure 3) [11,12,13,14]. Perforation, however, requires second level imaging, although bedside US in the operating room can easily help to stratify the patient by highlighting the presence of free fluid.

##### Urinary Tract

**Figure 4:**
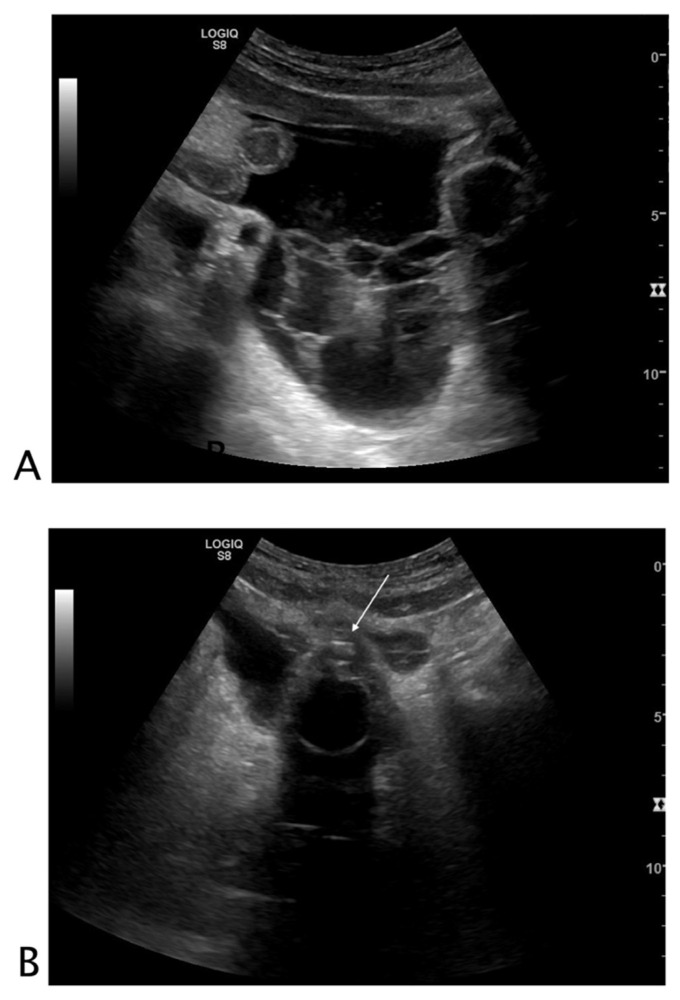
Black where it should not be. A 78-year-old male patient with abdominal pain who underwent robotic prostatectomy 20 days before. (**A**) Free fluid characterized by thick septa and inhomogeneous echogenicity was seen in all abdominal quadrants. (**B**) The bladder was empty and catheterized. Small hyperechogenic air bubbles were detected on the outer border of the bladder. A suspected diagnosis of post-surgical bladder perforation was formulated. The patient underwent CT with retrograde cystography without intravenous contrast for acute renal impairment, which confirmed the ultrasound diagnosis of bladder perforation. The patient underwent surgery. Final diagnosis: post-surgical bladder perforation.

Perforation of the collecting system may be spontaneous or due to pathology or intervention [15]. Urine leakage determines urinoma, urosepsis infection, and abscess. Rupture of the urinary collecting system is often caused by urinary obstruction. This can occur at any level and cause perinephric or retroperitoneal extravasation of urine [16,17]. Bladder perforation is reported in patients with previous bladder interventions, lower urinary tract obstruction, pelvic radiotherapy, inflammation, and malignancy. Spontaneous perforation is extremely rare (Figure 4) [18,19].

##### Gynecological Tract

Uterine rupture is a possible complication in all intrauterine procedures and may be associated with injury to surrounding blood vessels or viscera (bladder, bowel) [20]. One of the most frequent causes of non-traumatic hemoperitoneum is related to ectopic pregnancy or the rupture of an ovarian cyst for which pelvic US is the cornerstone of evaluation. Transabdominal US can be useful in the evaluation of early pregnancy by confirming intrauterine pregnancy and recognizing hemorrhage from ectopic pregnancy [21]. In suspected ectopic pregnancy, testing urine for pregnancy along with US scanning (preferably transvaginal) is the best method to obtain an accurate diagnosis and start appropriate management [21]. Abdominal free fluid can also be due to ovarian or fallopian tube torsion, in which the fluid effusion is accompanied by morphologic modifications of the gynecological tract affected due to abnormal blood flow [20,21,22].

##### Gallbladder

Gallbladder perforation diagnosis with US is tricky. The wall rupture is rarely seen, but indirect signs, such as pericholecystic fluid and focal areas of increased fat echogenicity, may help in the diagnosis [23,24].

##### Inflammatory Diseases

**Figure 5:**
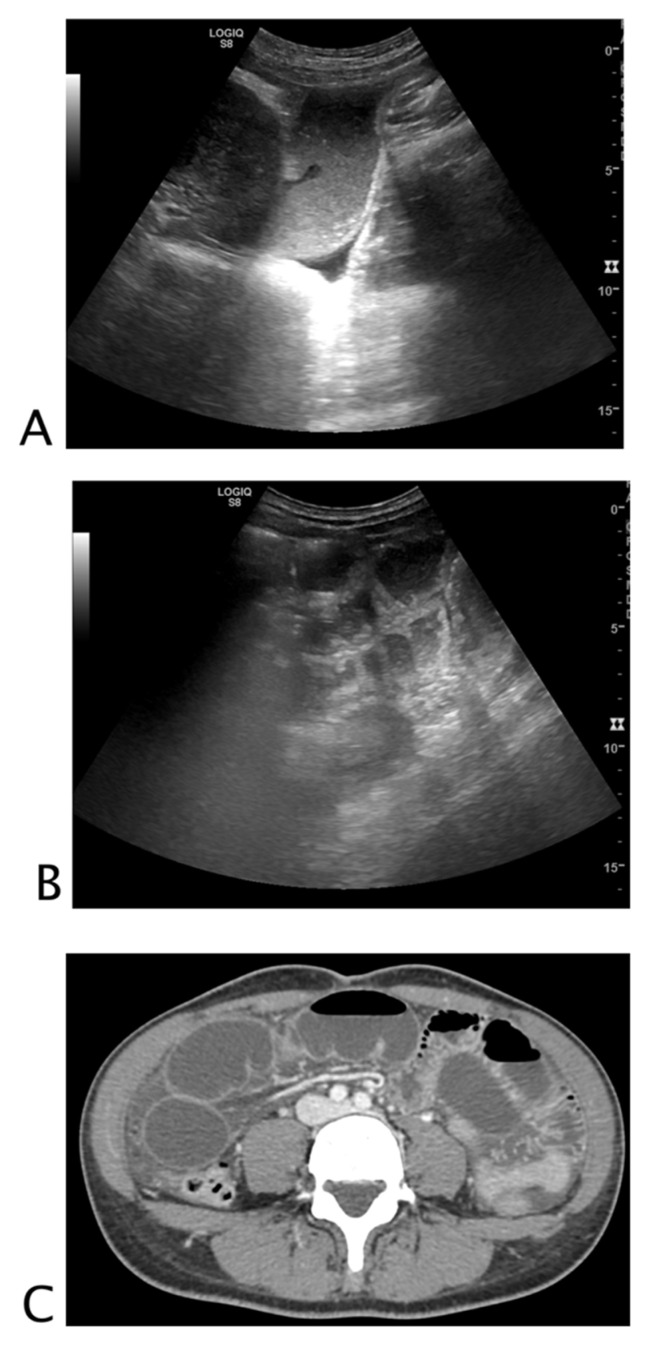
Black is too much, and it is where it should not be. A 68-year-old woman with abdominal distension, pain, and vomit. At ultrasound, (**A**) hypokinetic dilated small bowel loops were visualized on the right flank with thin parietal walls and poor evidence of valvulae, and free fluid was detected between bowel loops. On the left flank, (**B**) fluid between undilated bowel loops with regular representation of valvulae were detected. A suspected diagnosis of decompensated mechanical small bowel obstruction with fulcrum on the right flank was formulated. The patient underwent abdomino–pelvic CT; (**C**) pelvis that confirmed mechanical obstruction determined by adhesions on the right flank. Final diagnosis: decompensated small bowel obstruction.

Abdominal fluid in non-traumatic patients may be the expression of inflammatory diseases and infection and can be either free or organized. Free fluid around parenchymas and viscera is the expression of a complicated pathology or an extra-visceral extension of the disease where free fluid can stand next to the affected organ or organize into infected collections [25,26] (Figure 3 and Figure 5).

##### Intraparenchymal Fluid Lesions

**Figure 6:**
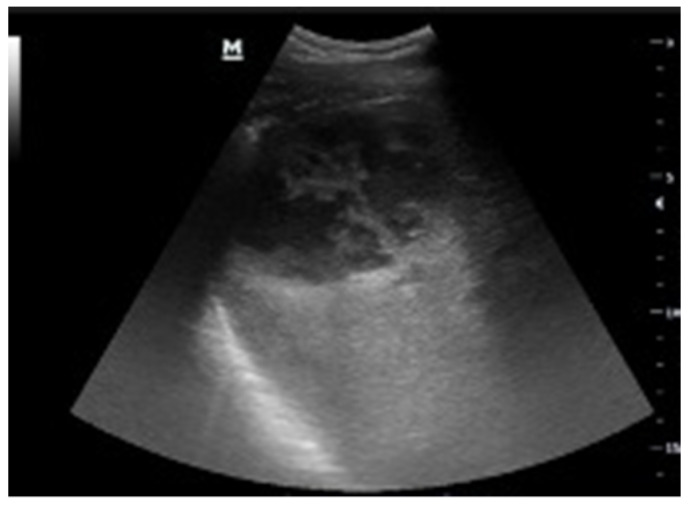
Black where it should not be. A 75-year-old man with recent history of low back pain treated with intramuscular anti-inflammatory therapy. He presented at the emergency department with pain in the left thigh on administration site. The ultrasound showed a corpuscle collection with the typical “swirl sign” during probe compression due to the movement of pus within the fluid collection. Final diagnosis: pyogenic liver abscess.

Apart from the presence of clearly benign uncomplicated anechoic cystic lesions, which may be due to infected cysts, either pre-existing or caused by infection, hematomas, and abscesses [5,27,28] (Figure 6), pseudoaneurysms are of variable origin; they may be congenital, post-traumatic and iatrogenic, or associated with systemic disease. Arteriovenous fistulae are rare complications of trauma and can be discovered incidentally years after the initial injury [28,29,30].

##### Soft Tissue

**Figure 7:**
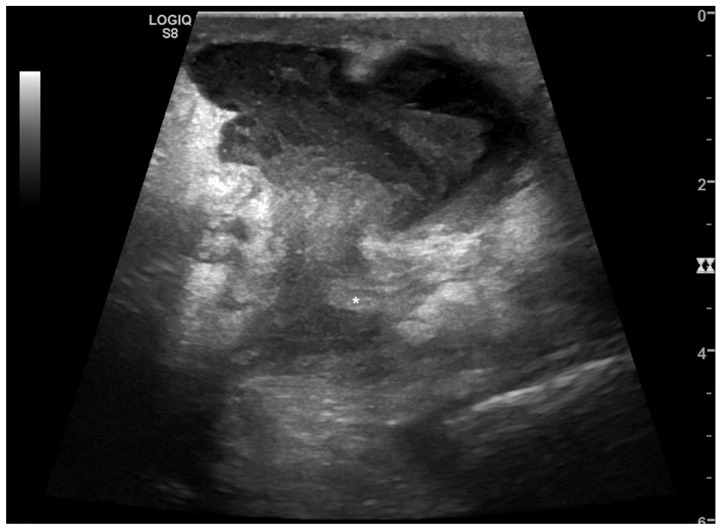
Black where it should not be. A 60-year-old male patient who underwent laparotomy gastrectomy with fever and abdominal pain. At ultrasound, a pluriloculated fluid collection was detected in the abdominal wall with a fistulous connection in the peritoneal cavity. No free fluid in the abdomen was detected. A suspected diagnosis of infected collection along the laparotomy suture was formulated. Final diagnosis: abscess along the laparotomy suture with peritoneal fistulous connection.

Superficial and intramuscular non-traumatic hematomas can occur secondary to invasive procedures or spontaneously, particularly in anticoagulated patients. Soft-tissue hematomas may be regular or irregular in shape with variable echogenicity, usually hyperechoic in the acute phase, and generally heterogeneous and hypoechoic later. An internal Doppler signal should be considered a sign of active bleeding [31,32]. Rectus sheath hematoma is an uncommon but dangerous cause of undifferentiated hypotension and abdominal pain in the emergency department (ED). Point-of-care US is a useful tool in its identification [33,34] (Figure 7).

**Figure 8:**
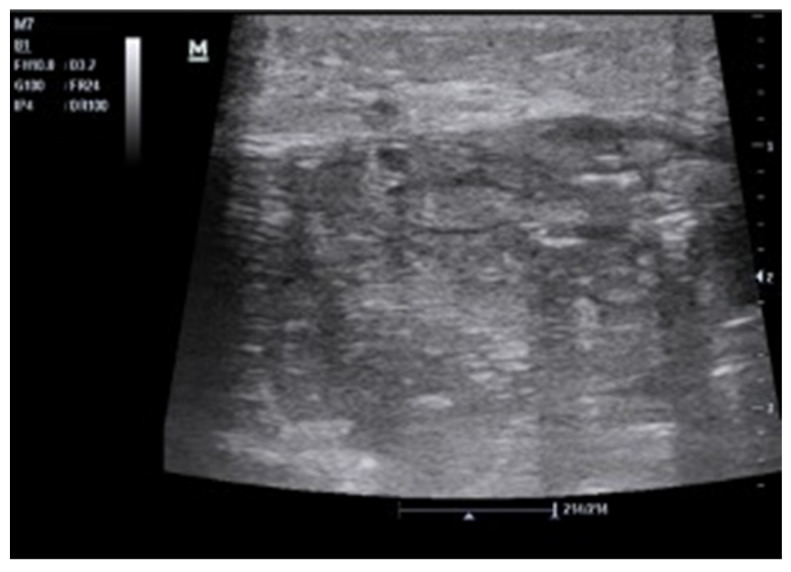
Black where it should not be. A 35-year-old man with a significant history of intravenous drug abuse presented with fever and local pain in the right forearm. The arm was warm and with tight skin. The ultrasound showed a necrotizing fasciitis with subcutaneous thickening, air, and fascial fluid. He underwent decompressive fasciotomy in association with large spectrum antibiotic therapy.

Soft tissue infections include cellulitis and abscesses. Cellulitis is characterized by non-compressible hyperechoic adipose tissue with non-loculated perifascial fluid effusion, usually defined as “cobblestoning”. An abscess can be organized/capsulated or ill-defined with inhomogeneous internal findings, ranging from anechoic to hyperechoic. The purulent material inside the abscess, when compressed by the US transducer, produces the typical “swirl sign” Clinical symptoms in addition to US findings and the patient’s history help in the differential diagnosis of hematoma, although at some stage even hematomas can become infected. The presence of air in soft tissues is highly suspicious for necrotizing fasciitis [33,34] (Figure 8).

### 2.2. Looking to See If There Is Too Much Black Where It Is Normally Present

Fluids are normally present in vessels, in the urinary tract, in the biliary system, and in the bowel. The presence of “too much fluid” suggests quite a wide range of pathologies (Table 2), which we report below:

#### 2.2.1. Vessels

A focal dilatation in an artery, with at least a 50% increase compared to its normal diameter, is defined as an aneurysm. Abdominal aortic aneurysms are defined as focal dilatations of the abdominal aorta that are 50% greater than the proximal normal segment or more than 3 cm in maximum diameter. Aortic CT and MRI are superior to US in the imaging of aortic disease location, morphology, extension, and complications [35,36]; however, bedside US is regarded as an effective diagnostic imaging modality of screening in the ED and its use has decreased mortality by 20% to 60% [37,38,39].

#### 2.2.2. Urinary Tract

US is the first imaging modality in non-complicated renal colic. It may help guide clinical suspicion and the need for further imaging in patients with less typical signs and symptoms [40,41,42]. The presence of hydronephrosis and urether dilation, along with the presence of renal and ureteral stones, represent the US signs of renal colic. The recently proposed diagnostic sign called “swinging kidney”, due to a characteristic antero-posterior “rolling” movement of the kidney, is another useful and new sign in diagnosis of nephrolithiasis [43]. Direct demonstration of the stone itself is not mandatory in the ED assessment. US allows for the detection of additional findings and alternate diagnosis [39,44].

#### 2.2.3. Biliary Tract

US is the best non-invasive method to evaluate patients with suspected biliary abnormalities [26,45]. Bile duct diseases result in dilatation of the intra- and/or extra-hepatic ducts. The width of the common bile duct in normal individuals does not exceed 6 mm measured at the crossing with the right hepatic artery. In elderly subjects and patients who have undergone cholecystectomy, the common bile duct can measure up to 8 mm in the absence of obstructive pathology. Usually, the intra-hepatic bile ducts are easily seen close to the hilum and do not normally exceed 2 mm. Visualization of dilated intrahepatic bile ducts centrally and peripherally—where they are usually not visible—is due to dilation. To increase diagnostic accuracy, the use of color Doppler can easily help to differentiate between dilated bile ducts and the vascular structure. Acute cholecystitis is caused by gallstones in 95% of cases whereas 5% of cholecystitis cases are acalculous. Although the most common abnormality is choledocholithiasis, the presence of biliary dilatation, even in the absence of direct stone identification, is still an independent predictor of the presence of gallstones [46,47,48]. US is the primary diagnostic tool for acalculous cholecystitis with hydrops, which is defined as distension greater than 8 cm longitudinally or 5 cm transversely. In this case, the black aspect of the gallbladder is much wider than it would normally be. Other US signs include a 3.5–4 mm (or more) thick wall (if the gallbladder is distended to at least 5 cm longitudinally, and the patient has no ascites or hypoalbuminemia), pericholecystic fluid, and/or sub-serosal edema [49].

#### 2.2.4. Bowel

US is highly accurate in confirming or excluding the presence of small bowel obstructions (sensitivity 92% and a specificity of 94%). US diagnostic criteria include the presence of dilated loops and abnormal peristalsis. Small bowel dilatation is defined as bowel diameter ≥ 2.5 cm measured from outer wall to outer wall [3,50,51] (Figure 5).

### 2.3. Looking for Black That Is Not Clearly Black

The diagnostic capability of US in characterizing fluid components is comparable to MR imaging. US can differentiate transudative, exudative, and complex fluid components such as debris and fluid/fluid levels [8,25,52]. The presence of heterogeneous fluids within the vessels, the urinary tract, or the biliary system can rapidly orient the diagnosis (Table 2). The following are the main US findings we can encounter.

#### 2.3.1. Vessels

**Figure 9:**
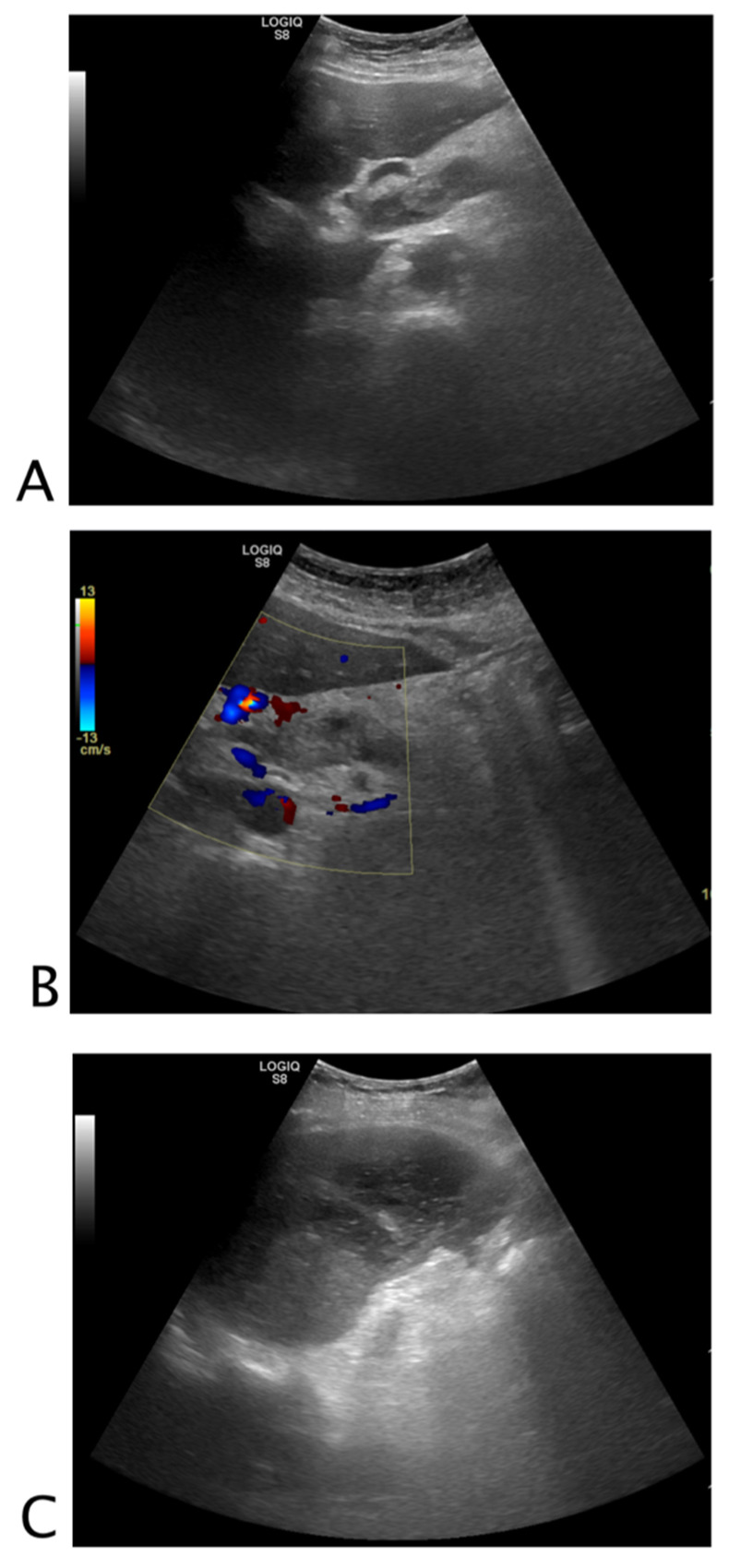
Black that is not clearly black. A 71-year-old female patient complaining of drug-resistant abdominal pain for the last three days. No history of malignancies or liver disease. At ultrasound, the portal vein was dilated and occupied by inhomogeneous echogenic material (**A**). The color Doppler evaluation confirmed the absence of flow in the portal vein (**B**). Hypoechogenic areas were detected in the spleen (**C**). A suspected diagnosis of portal vein thrombosis with associated spleen infarction was formulated. Final diagnosis: portal vein thrombosis in unknown thrombophilic disorder.

The presence of arterial intraluminal hypoechoic and heterogeneous material is highly suggestive of thrombus [53]. On the other hand, the presence of an intraluminal flap points to the diagnosis of arterial dissection. Venous US examination in emergency settings is widely used for the assessment of lower leg vein thrombosis, the most pivotal sign being the presence of a non-compressible vein, with the possible coexisting evidence of echogenic intraluminal material (thrombus) [35,54,55,56] (Figure 9).

#### 2.3.2. Urinary Tract

**Figure 10:**
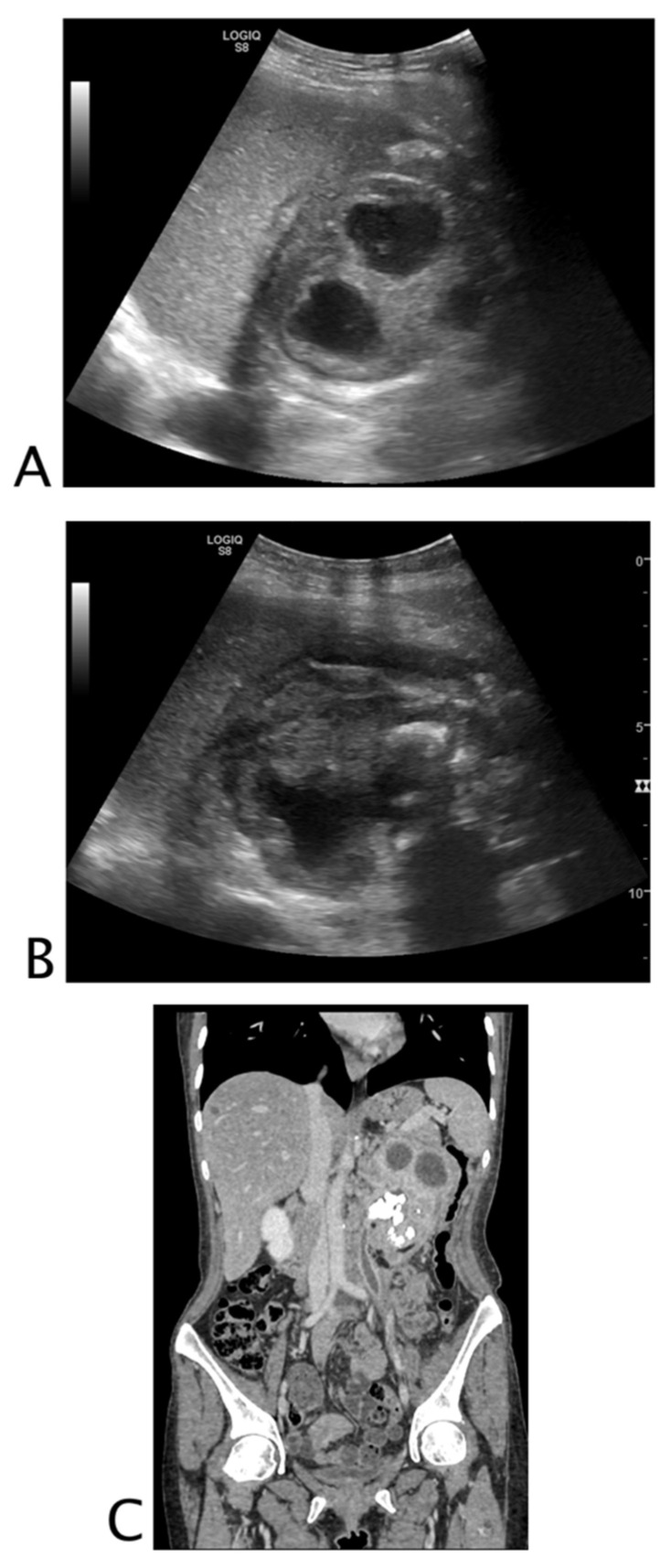
Black is too much, black is not clearly black, and black is where it should not be. A 47-year-old asthenic female patient with unintentional severe weight loss and left flank pain. At ultrasound, the left kidney was hardly recognizable; dilated upper calices with thick parietal walls and inhomogeneous urine (**A**) on the lower pole staghorn calculi were detected (**B**). Perinephric and subcapsular fluid was visualized. A suspected ultrasound diagnosis of complicated pyonephrosis was formulated and CT was performed in order to stage the pathology. (**C**) Final CT diagnosis: stage III xanthogranulomatous pyelonephritis with iliopsoas and lumbar muscle invasion.

The presence of hyperechoic spots within the urinary system is usually referred to calculi, although the presence of gas can mimic renal calculi. In a dilated calico–pelvic system and a distended bladder, the presence of intraluminal mass or material is easily appreciable. US findings include hydronephrosis with echogenic material in the collecting system, with a shifting urine debris. This finding has a 100% positive predictive value for suspected pyonephrosis and can thus ascertain the presence of urosepsis [25,57,58] (Figure 10).

#### 2.3.3. Biliary Tract

As in the case of the urinary tract, the presence of hyperechoic spots is also indicative of calculi in the biliary one [26,59]. Bedside US can evaluate for cholelithiasis, choledocholithiasis, and common bile duct dilatation with high specificity (94 to 100%) [60]. Regarding the gallbladder, gallstones appear as echogenic foci that cast an acoustic shadow with gravitational dependency; sludge is echogenic in appearance but does not cast an acoustic shadow. The sensitivity and specificity of ultrasonography for the detection of gallstones are approximately 84% and 99%, respectively [61]. Emphysematous cholecystitis is characterized by a highly echogenic collection of gas, which originates from the gallbladder wall or lumen, sometimes exhibiting gas bubbles that are seen rising from the dependent portion of the gallbladder towards the non-dependent side. This appearance has been described as similar to champagne bubbles rising in a glass [62].

## 3. Conclusions

Familiarity with the typical US appearance of fluid helps emergency clinicians to provide a timely diagnosis. The goal of our approach is not to introduce new data or protocols but to help clinicians to focus on fluid presence, easing the early identification of a large spectrum of abnormalities. This approach, starting from the three search-for-black levels, makes it possible to stratify the severity of the disease, improving the correct management, further imaging, and speeding up of an operative procedure. Based on what was previously described, we thus propose a targeted and methodical search for US black patterns in well-defined anatomical sites as a standard method, bearing in mind that, despite the high yield of such an approach, a secondary imaging is often mandatory. One of the limits of this paper is that it represents a teaching–reasoning concept, and the black approach needs to be verified prospectively. The list of clinical indications for abdominal ultrasound in non-traumatic abdominal emergencies is extremely wide and although the black approach can identify a wide spectrum of pathologies, it has to be focused on the patient’s symptoms and clinical suspects.

It is essential to guarantee an adequate US support in every emergency setting by ensuring training courses for the entire medical team.

## Figures and Tables

**Table 1:** Black protocol—look for black where it should not be.

**Right Upper Quadrant**	Morison pouch (hepatorenal recess), Liver tip (right paracolic gutter)
**Left Upper Quadrant**	Subphrenic space, Splenorenal recess, Spleen tip (left paracolic gutter)
**Pelvic**	Rectovesical pouch (male subjects) or, rectouterine/pouch of Douglas (female subjects)
**Between Bowel Loops**	Global view [3]
**Surgical Site**	Intrabdominal Soft tissue
**Intraparenchymal**	

**Table 2:** Black “approach”.

	Too Much Black	Black Where It Should Not Be	Black That Is Not Clearly Black
Urinary Tract	Renal colic Urinary obstruction	Ruptured urinary tract Complicated renal colic Urinoma Extrarenal diffusion of inflammatory/infectious process	Lithiasis Pyonephrosis Blood, clot/hemorrhage Hemorrhagic cyst
Bladder	Overdistension	Perforation Perivisceral inflammation	Cystitis Endoluminal mass Blood clot Lithiasis
Biliary tract	Biliary obstruction	Perivisceral inflammation Biloma	Intrabiliary lithiasis Cholangitis
Gallbladder	Cholecystitis	Perforation Perivisceral inflammation Perivisceral infected collection	Lithiasic cholecystitis Acalculous cholecystitis Emphysematous cholecystitis Mass
Small bowel	Obstruction	Perforation Obstruction Decompensated/complicated ileus	
Aorta	Aneurysm	Ruptured aneurysm	Thrombosis Dissection
Veins		Peri vessels oedema (phlebitis)	Thrombosis
Intraparenchymal		Infected cyst Infectious cyst Hematoma Abscess Aneurysm Pseudoaneurysm	

## Data Availability

Not applicable.
